# Normative values for musculoskeletal- and neuromotor fitness in apparently healthy Norwegian adults and the association with obesity: a cross-sectional study

**DOI:** 10.1186/s13102-016-0059-4

**Published:** 2016-11-18

**Authors:** Ingirid Geirsdatter Heald Kjær, Monica Klungland Torstveit, Elin Kolle, Bjørge Herman Hansen, Sigmund Alfred Anderssen

**Affiliations:** 1 Faculty of Health and Sports Science, The University of Agder, Postboks 422, 4604, Kristiansand, Norway; 2 Department of Sports Medicine, The Norwegian School of Sport Sciences, Sognsveien 220, Oslo, Norway

**Keywords:** Physical fitness, Mobility, Neuromuscular fitness, Muscular fitness, Muscular power, Reference values, Public health, Fatness

## Abstract

**Background:**

Up-to-date research on musculoskeletal- and neuromotor fitness (MSMF) is lacking. The aims of the present paper were to a) establish normative values of MSMF by gender and age, and b) to assess how much of the variance in MSMF can be explained by obesity in adults.

**Methods:**

A random selection of 726 Norwegians (20–65 years) participated in a national cross-sectional study. Muscular endurance, muscular strength, explosive power, flexibility and balance were assessed in addition to waist circumference (WC).

**Results:**

Females displayed significantly higher scores compared to males on muscular endurance of the back extensors and on the flexibility tests (*p* < 0.001). Males displayed significantly higher scores than females (*p* < 0.001) on handgrip strength, modified push-ups, and explosive power. An inverse association was found between age and all MSMF scores for females (Beta:−0.06–(−0.92), *p* ≤ 0.044) and males (Beta:−0.15–(0.91), *p* ≤ 0.006), where younger participants displayed higher test scores on all MSMF tests, compared to older participants. Furthermore, participants showing higher scores on WC displayed lower scores on the following MSMF tests for both females and males: muscular endurance of the back extensors, balance, flexibility of the shoulder, and explosive power (*p* < 0.001). Additionally, male participants with higher WC scores showed lower scores on muscular endurance of the upper body and flexibility of the hamstrings compared to males with lower WC scores (*p* < 0.001).

**Conclusions:**

The data provide normative values of MSMF for adults based on age and gender, and support an inverse relationship of MSMF to age and WC.

**Electronic supplementary material:**

The online version of this article (doi:10.1186/s13102-016-0059-4) contains supplementary material, which is available to authorized users.

## Background

Cardiorespiratory fitness and muscular strength seem to provide unique and important benefits to the prevention and treatment of cardiovascular disease and mortality in addition to several other health and fitness variables [1, 2]. Both the American College of Sports Medicine (ACSM) position stand on the Quantity and quality of exercise for developing and maintaining cardiorespiratory, musculoskeletal, and neuromuscular fitness in apparently healthy adults [3] and recently published Nordic nutrition recommendations integrating nutrition and physical activity [2] emphasize the importance of combining cardiorespiratory exercise with muscular strength, flexibility and neuromotor exercise (e.g. balance, coordination, agility and gait) as essential in preventing disease, in addition to improving health, and quality of life.

Physical fitness decreases with increasing age at varying rates, depending on lifestyle and physical health earlier in life [3–5]. Additionally, clear gender differences have been reported not only in muscular strength, but also in muscular endurance, balance, flexibility and explosive power, though some of the results are inconsistent. The literature indicate that males display significantly higher mean scores on muscle strength [6, 7] and balance [8]. Females seem to generally display higher scores on flexibility compared to males [9–11]. Inconsistency has been found for gender differences in muscular endurance of the back [12] and for flexibility of the shoulder [8, 11, 13].

Few studies have investigated the relationship between musculoskeletal- and neuromotor fitness (MSMF) and obesity. Most studies have reported an inverse relationship between various aspects of MSMF and obesity assessed by body mass index (BMI) or waist circumference (WC) [6, 14–16], where scores on MSMF decline with increasing BMI or WC values or categories, while some studies have found positive relationships between handgrip strength and BMI [14, 15].

There is a clear lack in up-to-date normative data on field based MSMF assessing muscular strength, muscular endurance, explosive power, flexibility and balance. Most of the present studies have assessed almost solely hand grip strength [6, 17–21]. The latest published Scandinavian data on MSMF are a Norwegian regional study of 566 adults and elderly (20–94 years) [19] and a national Danish study of 3471 males and females (19–72 years) [6]. However, these Scandinavian studies cover only three aspects of MSMF (pinch grip, hand grip strength and lower limb extension power). The latest published normative data on field based MSMF tests covering various elements are those of Rikli and Jones [10] studying older adults aged 60–94 years (*N* = 7183), and data published by the ACSM [9], based on Canadian normative values published in 2000 [22].

Based on the previously highlighted issues, the aims for this study were to a) establish normative values of MSMF by age and gender covering a wide range of MSMF, and b) to assess how much of the variance in MSMF can be explained by obesity in a sample of adult Norwegian males and females aged 20–65 years.

## Methods

### Study design and sample

The present study is part of a national, multicenter cross-sectional study [23], and has been approved by the Regional Committee for Medical Ethics (REK Sør-Øst B, S-08046b) and the Norwegian Social Science Data Services. In order to sign up for participation, all participants signed and returned a written consent form.

A total of nine regional test centers nationwide in Norway participated in the two separate data collecting phases of the study. The initial phase was conducted in 2008 and assessed physical activity level using the ActiGraph GT1M accelerometer (ActiGraph, LLC), as well as demography, educational level, nutritional status, tobacco use, physical activity level and correlates for physical activity amongst other things through a questionnaire [24]. Initially, a random representative sample of 11,515 individuals, aged 20–85 years was drawn from the Norwegian National Population Registry and invited to participate. A total of 3800 agreed to participate in the initial data collection phase, where valid data from 3464 males (*n* = 1614) and females (*n* = 1850) were included. The second phase was conducted in 2009 and assessed physical fitness, including assessment of maximal oxygen consumption (VO_2max_), musculoskeletal- and motor fitness, blood pressure, lung capacity, and body composition [23]. A random selection of 1930 participants from the initial phase, were invited to take part in the second phase, where 463 males and 441females participated. For this paper, only the adult participants, aged 20.0–64.9 years were included (*N* = 726, F:350, M:376).

### Methods and procedures

The following MSMF tests were included in this study and carried out in the following order; Muscular endurance of the upper body was measured by the static back extension test (SBE) [25]. The handgrip strength test (HGS) [26, 27] recorded muscular strength of the hand. Neuromuscular fitness was measured by the one leg standing test (OLS). The modified push-ups test (MPU) [25] assessed muscular dynamic endurance and ability to stabilize the upper body. Alternately, if the participants could not complete the ordinary MPU test, the MPU test was carried out on the knees (MPUK). The sit and reach test (SR) [28] recorded the flexibility of the hamstring musculature, and the back scratch test (BSC) [29] measured flexibility in the shoulder joint. In addition, explosive power in the lower extremities was measured by the vertical jump test (VJ) [25] and the explosive power on a power platform test (EPP) (HurLabs Force platform). Data on VO_2max_ are published elsewhere [23]. For further elaboration on the tests used to assess MSMF, see Additional file 1: Appendix.

A health risk assessment was conducted prior to all physical fitness assessments and consisted of a questionnaire comprising elements of general health status. The health risk assessment was conducted in order to eliminate potential participants at risk, though none were considered to be at risk. All participants went through a 10–15 min warm-up before the MSMF tests were conducted. For all tests, the instructor demonstrated the test-procedure before the participants conducted the test. All measures were conducted by trained instructors following a detailed test protocol and all measuring instruments were calibrated.

### Measures of adiposity

Height was measured to the nearest centimeter (cm) using a stadiometer while the participant was standing upright with the heels touching the wall, without shoes. Body weight was measured to the nearest 0.1 kilogram (kg) with minimal clothing, using Seca weight scales. BMI was calculated (kg/m^2^). The BMI values were further grouped based on cut-off points developed by the World Health Organization (WHO) [30], where those with BMI values < 18.5 kg/m^2^ were categorized as underweight, between 18.5 and 24.9 kg/m^2^ were categorized as normal weight, between 25.0 and 29.9 kg/m^2^ were categorized as overweight and those with BMI values ≥ 30 kg/m^2^ were categorized as obese. WC was measured at the mid-point between the upper most lateral part of the iliac crest and the lowest most lateral point of the ribcage using a measuring band, where the mean of two measures was recorded to the nearest half centimeter. The WC values were grouped based on cut-off values developed by the WHO [30]. Males and females with WC values of ≥ 94 cm and ≥ 80 cm, respectively, were categorized as abdominally overweight and males and females with WC values ≥ 102 cm and ≥ 88 cm, respectively, were categorized as abdominally obese.

### Statistics

The collected data were analyzed using IBM SPSS Statistics 22 (IBM Corporation, Route, Somers, NY, USA). The mean of the two BSC tests for left and right arm resulted in a mean BSC variable, which is the only BSC variable used in this study. Furthermore, the OLS test and the OLS blinded test were summed and the OLSsum variable was created which is the only OLS variable used in this study. As only six female participants were classified as underweight, and the lowest BMI score among these was 18.0 kg/m^2^, the underweight participants were grouped in the normal weight category in the analyses. All the MSMF tests were considered normally distributed based on histogram distribution, except for the OLSsum test which peaked markedly at 60 s.

The normative values were given by mean ± standard deviation (SD), and quartile 1 (Q1) to quartile 3 (Q3), displayed by gender and 10-year age groups. An independent samples *t*-test was used to detect gender differences on all MSMF tests, with the exception of the OLSsum tests where a Mann Whitney *U* test was used. A regression analysis was run in order to analyze how much of the variance in MSMF could be explained by age and obesity. As WC and BMI correlated significantly (Pearson *r* = 0.796, *p* = 0.001), and as BMI has been more closely related to muscle mass and WC more closely related to fat mass [14], we chose WC as the main indicator for obesity in the standard multiple regression analysis. The results from the regression analyses between age and MSMF, and between WC and MSMF, were thereby given by Beta and beta of the z-score (Beta Z) in brackets, *p*-value and 95 % confidence interval (95 % CI), displayed by gender. A statistical significance value of probability was set to *p* ≤ 0.05.

## Results

Sample characteristics are presented in Table 1. A total of 58 % (F:61.4 %, M:54.8 %) of the sample had completed higher education (College/University). Furthermore, based on BMI, a total of 37.7 % (F:27.1 %, M:46.6 %) of the sample were classified as overweight and 13.5 % (F:14.3 %, M:12.8 %) as obese. In addition, based on WC, 27.4 % (F:28.0 %, M:26.8 %) were abdominally overweight and 28.1 % (F:35.5 %, M:21.1 %) were abdominally obese (Table 1).

**Table 1:** Sample characteristics

Characteristic	Females		Males	
	n	Mean ± SD	n	Mean ± SD
Age (years)	350	46.0 ± 11.5	376	46.3 ± 11.7
Weight (kg)	350	70.5 ± 13.2**	376	85.2 ± 12.0**
Height (m)	350	1.67 ± 0.06**	376	1.80 ± 0.06**
BMI (kg/m^2^)	350	25.2 ± 4.4**	376	26.3 ± 3.4**
Overweight	95	27.1 %*	179	47.6 %*
Obese	50	14.3 %*	48	12.8 %*
WC (cm)	347	84.7 ± 11.3**	373	94.0 ± 10.4**
Abdominally overweight	97	28.0 %*	100	26.8 %*
Abdominally obese	123	35.5 %*	79	21.1 %*
Educational level				
< High school	29	8.4 %	25	6.8 %
High school	105	30.3 %	141	38.4 %
< 4 years university	94	27.1 %	88	24.0 %
≥ 4 years university	119	34.3 %	113	30.8 %

Sample characteristics given as number (*n*) and mean ± standard deviation (*SD*), displayed by gender. Weight groups (BMI and WC) and educational level given as percentage, displayed by gender

*Abbrevations*: *NS* Non-significant

*p < 0.001 for gender differences in prevalence distribution between weight categories

**gender difference *p* < 0.001

### MSMF–status and normative values

Normative values on all the MSMF tests are displayed in Table 2. The total sample displayed significant gender differences on all MSMF tests (*p* < 0.001), except for the OLSsum (Table 2). Males displayed significantly higher scores on HGS (F:32.1 kg, M:58.8 kg, *p* < 0.001), MPU (F:8.5 rep, M:12.1 rep, *p* < 0.001), VJ (F:24.7 cm, M:38.0 cm, *p* < 0.001) and EPP (F:19.5 cm, M:29.3 cm, *p* < 0.001), while females displayed significantly higher scores on SBE (F:90.9 s, M:74.3 s, *p* < 0.001), SR (F:24.3 cm, M:18.9 cm, *p* < 0.001) and BSC (F: −2.3 cm, M: −8.1 cm, *p* < 0.001).

**Table 2:** MSMF–status and normative values

Test	Age group (years)	Number	Females		Number	Males		Gender diff. *p*-value
			Mean ± SD	Q1–Q3		Mean ± SD	Q1–Q3	
Static back extension SBE (sec)	All ages	338	90.9 ± 49.5	58.0–120.0	360	74.3 ± 38.3**	52.0–96.8	**
	20.0–29.9	36	101.1 ± 41.9	63.8–124.8	39	86.0 ± 37.0	58.0–120.0	NS
	30.0–39.9	73	110.6 ± 49.3	68.5–142.5	78	84.5 ± 30.9**	60.8–107.0	**
	40.0–49.9	101	83.4 ± 42.7	57.5–110.0	96	76.0 ± 41.4	50.3–90.0	NS
	50.0–59.9	85	82.8 ± 54.7	43.9–111.5	94	66.6 ± 33.6*	48.0–80.5	*
	60.0–64.9	43	82.3 ± 51.3	45.0–110.0	53.0	61.1 ± 45.0*	32.5–77.5	*
Handgrip strength HGS (kg)	All ages	347	32.1 ± 6.0	28.0–36.0	375	54.8 ± 9.5**	48.0–60.0	**
	20.0–29.9	36	33.4 ± 6.0	29.3–39.0	40	58.0 ± 10.3**	51.0–64.0	**
	30.0–39.9	73	33.8 ± 6.0	30.0–38.0	78	58.8 ± 9.6**	52.0–64.0	**
	40.0–49.9	104	33.1 ± 5.6	30.0–36.0	100	55.8 ± 8.1**	50.0–61.0	**
	50.0–59.9	89	30.7 ± 6.1	27.0–34.0	102	53.4 ± 7.8**	48.0–59.0	**
	60.0–64.9	45	28.8 ± 5.2	25.0–32.0	55	47.9 ± 9.8**	42.0–55.0	**
One leg standing summed OLSsum (max 120 s)	All ages	349	57.4 ± 22.7	44.5–68.0	374	55.7 ± 22.9	42.0–67.0	NS
	20.0–29.9	37	72.7 ± 23.4	63.5–76.5	40	66.4 ± 13.4	62.3–74.5	NS
	30.0–39.9	73	67.5 ± 19.7	64.0–74.0	78	66.6 ± 16.2	63.0–72.53	NS
	40.0–49.9	103	58.9 ± 17.2	55.0–68.0	100	60.2 ± 21.5	62.0–68.8	NS
	50.0–59.9	89	50.7 ± 21.7	34.0–66.0	101	50.3 ± 21.2	34.0–64.0	NS
	60.0–64.9	47	39.5 ± 23.2	15.0–63.5	55	33.8 ± 24.2	11.0–63.0	NS
Modified push-ups MPU (no/40 s)	All ages	150	8.5 ± 4.3	6.0–11.3	342	12.1 ± 4.7**	9.0–15.0	**
	20.0–29.9	23	10.3 ± 4.2	7.0–13.0	39	14.2 ± 3.2**	12.0–16.0	**
	30.0–39.9	44	9.5 ± 4.4	7.0–13.0	75	14.3 ± 5.0**	11.0–17.0	**
	40.0–49.9	47	7.7 ± 3.9	6.0–10.0	96	12.6 ± 4.7**	9.0–16.0	**
	50.0–59.9	25	7.8 ± 4.5	5.0–10.5	90	10.5 ± 4.0*	8.0–13.0	*
	60.0–64.9	11	5.1 ± 3.0	2.0–7.0	42	8.4 ± 3.4*	6.0–10.0	*
Modified push-ups on knees MPUK (no/40 s)	All ages	196	7.9 ± 4.4	5.0–11.0	-	-	-	-
	20.0–29.9	14	6.6 ± 5.2	0.0–11.5	-	-	-	-
	30.0–39.9	32	8.9 ± 4.2	6.3–11.8	-	-	-	-
	40.0–49.9	56	9.0 ± 4.9	6.0–12.0	-	-	-	-
	50.0–59.9	61	7.3 ± 4.0	5.0–10.0	-	-	-	-
	60.0–64.9	33	6.8 ± 3.6	4.0–9.0	-	-	-	-
Sit and reach SR (cm)	All ages	348	24.3 ± 13.2	17.3–33.9	373	18.9 ± 12.0**	11.0–28.0	**
	20.0–29.9	37	24.4 ± 14.8	19.0–33.8	40	20.8 ± 11.0	15.0–28.4	NS
	30.0–39.9	73	29.0 ± 14.3	22.0–38.5	78	21.1 ± 12.2**	13.0–31.6	**
	40.0–49.9	103	22.3 ± 13.1	15.0–31.5	100	20.2 ± 12.6	10.6–30.4	NS
	50.0–59.9	88	23.8 ± 10.6	16.0–30.9	100	16.5 ± 11.8**	8.0–25.5	**
	60.0–64.9	47	22.1 ± 13.1	14.5–32.0	55	16.8 ± 10.9*	9.0–25.0	*
Back scratch BSC (cm)	All ages	343	−2.3 ± 8.5	−7.5–4.3	366	−8.1 ± 11.2**	−15-5–0.0	**
	20.0–29.9	37	1.1 ± 7.8	−3.9–6.4	40	−0.2 ± 8.9	−3.9–4.8	NS
	30.0–39.9	71	1.8 ± 8.0	−1.5–7.0	77	−3.1 ± 10.0**	−8.5–3.7	**
	40.0–49.9	101	−2.2 ± 8.7	−7.8–3.9	98	−7.7 ± 10.2**	−15.1–(−0.5)	**
	50.0–59.9	87	−5.2 ± 7.2	−9.0–0.0	99	−11.0 ± 10.0**	−18.5–(−4.5)	**
	60.0–64.9	47	−5.8 ± 8.0	−11.0–0.0	52	−16.6 ± 10.9**	−23.0–(−9.8)	**
Vertical jump VJ (cm)^	All ages	237	24.7 ± 7.1	20.0–29.2	247	38.0 ± 8.5**	32.0–45.0	**
	20.0–29.9	16	29.8 ± 5.8	25.5–32.8	25	44.7 ± 9.1**	40.8–49.5	**
	30.0–39.9	50	29.8 ± 6.4	26.0–34.0	51	43.1 ± 7.4**	38.5–48.0	**
	40.0–49.9	84	25.8 ± 6.1	22.0–29.5	69	38.6 ± 7.5**	34.8–43.5	**
	50.0–59.9	57	20.7 ± 5.6	16.5–24.3	69	35.9 ± 6.1**	31.5–39.0	**
	60.0–64.9	30	18.6 ± 5.3	15.6–23.3	33	28.4 ± 5.4**	24.3–31.5	**
Explosive power on power platform EPP (cm)^	All ages	191	19.5 ± 5.1	15.9–23.0	191	29.3 ± 7.2**	24.1–34.4	**
	20.0–29.9	23	24.2 ± 3.7	21.8–26.4	24	36.2 ± 4.8**	33.6–39.3	**
	30.0–39.9	39	22.1 ± 4.7	19.9–24.8	37	34.3 ± 5.8**	30.6–38.3	**
	40.0–49.9	55	19.7 ± 4.1	17.4–22.7	51	29.5 ± 6.2**	23.8–32.6	**
	50.0–59.9	50	17.1 ± 3.8	14.1–20.1	52	26.3 ± 5.7**	22.6–30.1	**
	60.0–64.9	24	15.4 ± 5.4	11.0–17.4	27	21.8 ± 4.3**	18.1–24.7	**

Normative values for all the MSMF scores displayed by test, age group, number (*n*), mean ± standard deviation (*SD*), quartile 1 (*Q1*) and quartile 3 (*Q3*)

- too few cases for further analysis (n for all ages = 21), *p < 0.05 for gender differences, **p < 0.001 for gender differences, *NS* Not statistically significant

^ Vertical jump and explosive power on a power platform were only conducted at selected test-centers due to availability of resources and equipment

All MSMF tests were inversely associated with age for both females (*p* ≤ 0.044) and males (*p* ≤ 0.006), where younger participants scored significantly higher on all MSMF tests, compared to older participants (Table 3). The largest reduction in MSMF test scores related to age for females was found in the OLSsum (Beta Z = −0.04), where test scores declined with 0.91 s for every 1 year increase in age (*p* < 0.001). HGS, MPUK and SR tests displayed the least reduction in test scores related to age in females (Beta Z for HGS, MPUK and SR = −0.01). For males, the largest decline in test scores for MSMF associated with age was found in the EPP test (Beta Z = −0.05). A 1 year increase in age was associated with a reduction in EPP test scores of 0.40 cm (*p* < 0.001). The SR scores declined least in relation to age (Beta Z = −0.01) for males.

**Table 3:** MSMF in relation to age (years)

	Females			Males		
	Beta (Beta Z)	*P*-value	95 % CI	Beta (Beta Z)	*P*-value	95 % CI
Static back extension SBE (sec)	−0.91 (−0.02)	<0.001	−1.36–(−0.46)	−0.72 (−0.02)	<0.001	−1.05–(−0.39)
Handgrip strength HGS (kg)	−0.14 (−0.01)	<0.001	−0.19–(−0.08)	−0.28 (−0.02)	<0.001	−0.36–(−0.20)
One leg standing summed OLSsum (max 120 s)	−0.91 (−0.04)	<0.001	−1.10–(−0.73)	−0.91 (−0.04)	<0.001	−1.08–(−0.73)
Modified push-ups MPU (no/40 s)	−0.13 (−0.03)	<0.001	−0.18–(−0.07)	−0.16 (−0.03)	<0.001	−0.20–(−0.12)
Modified push-ups on knees MPUK (no/40 s)	−0.06 (−0.01)	0.044	−0.11–(−0.00)	-	-	-
Sit and reach SR (cm)	−0.13 (−0.01)	0.036	−0.25–(−0.01)	−0.15 (−0.01)	0.006	−0.25–(−0.04)
Back scratch BSC (cm)	−0.26 (−0.03)	<0.001	−0.33–(−0.18)	−0.42 (−0.04)	<0.001	−0.51–(−0.33)
Explosive power on a power platform EPP (cm)	−0.24 (−0.03)	<0.001	−0.29–(0.19)	−0.40 (−0.05)	<0.001	−0.47–(−0.54)
Vertical jump VJ (cm)	−0.35 (−0.03)	<0.001	−0.42–(−0.29)	−0.39 (−0.04)	<0.001	−0.47–(−0.31)

A regression analysis for all the MSMF tests against age (years), given by Beta (Beta Z), *p*-value and 95 % CI, displayed by gender, where Beta Z is the beta of the z-score of each MSMF test

- not analyzed due to low sample size (*n* = 21)

### MSMF in relation to WC

WC was found to be inversely associated with scores on SBE (Beta = F: −1.75, M: −1.37,), OLS (Beta = F: −0.58, M: −0.55), BSC (Beta = F: −0.37, M: −0.52), and both explosive power tests, VJ (Beta = F: −0.19, M: −0.26) and EPP (Beta = F: −0.14, M: −0.34) for both genders (*p* < 0.001), where higher WC scores were associated with lower MSMF test scores. Additionally, higher WC scores were related to lower scores on MPU (Beta = −0.19) and SR (Beta = −0.29) in males (*p* < 0.001) (Table 4). For females, the largest change in MSMF by each one cm increase in WC, was found for SBE (Beta Z = −0.04). SBE decreased by −1.75 s per one cm increase in WC in females (*p* < 0.001). The equivalent for males was found for BSC (Beta Z = −0.05), which decreased by −0.52 cm per one cm increase in WC (*p* < 0.001).

**Table 4:** MSMF in relation to WC

	Females			Males		
	Beta (Beta Z)	*P*-value	95 % CI	Beta (Beta Z)	*P*-value	95 % CI
Static back extension SBE (sec)	−1.75 (−0.04)	<0.001	−2.19–(−1.32)	−1.37 (−0.03)	<0.001	−1.73–(−1.02)
Handgrip strength HGS (kg)	0.04 (0.00)	0.156	−0.02–0.10	−0.01 (0.00)	0.849	−0.10–0.08
One leg standing summed OLSsum (max 120 s)	−0.58 (−0.03)	<0.001	−0.78–(−0.37)	−0.55 (−0.02)	<0.001	−0.77–(−0.33)
Modified push-ups MPU (no/40 s)	−0.05 (−0.01)	0.148	−0.11–0.02	−0.19 (−0.04)	<0.001	−0.23–(−0.14)
Modified push-ups on knees MPUK (no/40 s)	−0.04 (−0.01)	0.125	−0.10–(0.01)	-	-	-
Sit and reach SR (cm)	−0.10 (−0.01)	0.117	−0.22–0.03	−0.29 (−0.02)	<0.001	−0.40–(−0.18)
Back scratch BSC (cm)	−0.37 (−0.04)	<0.001	−0.44–(−0.30)	−0.52 (−0.05)	<0.001	−0.62–(−0.42)
Explosive power on a power platform EPP (cm)	−0.14 (−0.02)	<0.001	−0.20–(−0.08)	−0.34 (−0.04)	<0.001	−0.43–(−0.26)
Vertical jump VJ (cm)	−0.19 (−0.02)	<0.001	−0.26–(−0.11)	−0.26 (−0.03)	<0.001	−0.35–(−0.16)

A regression analysis for all the MSMF tests against waist circumference (WC), given by Beta (Beta Z), *p*-value and 95 % CI, displayed by gender, where Beta Z is the beta of the z-score of each MSMF test

- not analyzed due to low sample size (*n* = 21)

## Discussion

Our results display clear gender differences on all MSMF tests, except for the OLSsum and MPUK tests. Increasing age was associated with lower MSMF test scores for both genders. Furthermore, higher WC scores were associated with lower scores on SBE, OLS, BSC and both explosive power tests for females and males, in addition to MPU and SR for males.

### MSMF status

A national Danish study of 3471 females and males (19–72 years) [6], displayed mean HGS scores which were similar to those found in the present study. Compared to a regional Norwegian study of 566 adults and elderly (20–94 years) [19], the present study found mean HGS scores by 10 year age groups which were primarily similar, except for males in the age group of 50.0–59.9 years. Males in their 50s displayed markedly lower scores on HGS in the study by Nilsen et al. [19], compared to our study. Whether or not the difference in HGS test scores reflect regional differences, differences in recruitment procedures or differences in sample characteristics in this specific age group is therefore unclear, though they should be considered as possible explanatory factors as the differences in this specific age group for males are noteworthy. The study by Aadahl, Beyer, Linneberg, Thuesen, and Jorgensen [6], however displayed mean HGS scores which were similar to those found in the present study.

Compared to normative values for HGS published by the ACSM [9], the mean HGS scores from our study were within or above the cut-off for *average* in all age groups. The normative HGS scores presented by the ACSM are based on the sum of the average measures from both the right and the left hand grip, whereas our study recorded the highest HGS score for one hand. As an average score would be expected to be lower compared to a peak score, the ACSM scores would be expected to be lower compared to the ones reported in this study. Hence, the difference in assessment protocol may imply an overestimation of the HGS status in our study.

The ACSM [9] also published age based normative values for hamstrings flexibility by SR, where mean SR values from the present study reveal scores within the category of *needs improvement* and *fair*. The assessment protocol for SR recommended by the ACSM, differs slightly from that used in our study in that they allow for flexion of the neck when leaning forward toward the measuring band, while our study’s protocol instruct the participants to keep a straight back. Whether this difference in assessment method may be a significant explanatory factor for the observed differences between the present study and the normative values by the ACSM is unknown, thus prone for speculation. Furthermore, the reference data published by the ACSM [9] is based on a study on a representative sample Canadian adults (*N* = 571, 15–65 years) dated prior to 2000 [22]. The time difference in the studies data collection may question the comparability of the categorization of test scores, though the link between various MSMF measures and health outcomes investigated in the study by Payne, Gledhill, Katzmarzyk, Jamnik, and Ferguson [22] is believed to remain unchanged. For the remaining tests, we found no further studies we considered comparable in design, methods or sample.

The spread in scores on the flexibility tests (BSC and SR) and the muscular endurance tests (MPU, MPUK and SBE) were large, not only for the mean of the entire sample (20.0–64.9 years), but also within each 10-year age group and for both genders. Whether or not this observed variation in test scores is attributable to measuring methods or a naturally large population variation on these specific MSMF properties, is uncertain, though the reported variability in test scores should be taken into consideration when interpreting and applying the normative values. Further research is needed in order to address variability in MSMF.

### MSMF and gender

Although inconsistencies in gender differences have been found in previous studies, clear gender differences were found within the different MSMF scores in our study. In accordance with the present findings, other studies have also reported male superiority in muscular strength [7, 17, 19, 20]. Even though our findings display female superiority on the SBE test, muscular endurance by SBE seems to be a measure of musculoskeletal fitness in which gender differences are not as clear as other aspects of musculoskeletal fitness [12]. Haizlip, Harrison, and Leinwand [31] clearly state that muscular endurance is a measure of muscular fitness in which females are superior to males, due to the larger number of type I muscle fibers characterized by slow oxidative metabolism. However, males showed significantly higher mean scores on muscular endurance by the MPU test in the present study. Furthermore, markedly more males (*n* = 342), than females (*n* = 150) completed the MPU test, whereas markedly more females (*n* = 196), than males (*n* = 21) completed the MPUK test. Whether or not the observed inequalities between genders concerning choice of test is related to the registered male superiority in muscular endurance of the upper body, or other factors is unclear, though noteworthy for future testing of and research on muscular endurance. With the exception of Bø and Hagen’s [8] findings of male superiority on flexibility of the shoulder, the remaining literature on flexibility of the shoulder seem to indicate female supremacy in flexibility [11, 13], which is in accordance with our findings, though data is sparse. Manire, Kipp, Spencer and Swank [32] mention the possibility of females’ longer m. hamstrings muscle length, as a possible explanatory factor related to females superiority in m. hamstring flexibility, though they clearly state that more research is needed in order to elaborate the mechanisms underlying the difference in flexibility between genders. No difference in score on the OLS test was found in this study, however in previous studies, males have shown better scores on balance compared to their female peers [8, 10]. The lack of data in this field and the lack of agreement in the studied literature, give little room for resolution.

### MSMF and age

An increase in age was associated with lower test scores on all MSMF aspects. Muscular strength across the major joints in the body has previously been shown to decrease with increasing age [6, 7, 19, 20]. The age dependency has been shown to vary from movement to movement, though most studies report increasing decline in muscular strength from 40 to 50 years of age [6, 7, 19, 20]. Furthermore, the age related declines in flexibility, muscular endurance and explosive power found in our study are supported by previous findings [6, 8, 12]. The present study, however, is cross sectional and cannot imply age related changes in MSMF, or the subsequent cause of differences in scores on the various MSMF tests observed between age groups.

### MSMF and obesity

Increased WC values were found to be associated with decreased scores on SBE, OLS, BSC, EPP and VJ for both genders, in addition to MPU and SR for males. According to the Beta of the z-scores (Beta Z) females decreased most in test score on the SBE and BSC tests per one cm increase in WC and least on both explosive power tests. Males decreased most in test score on the BSC test and least on the OLSsum, SR and VJ per one cm increase in WC. The increased WC scores associated with decreased test scores on BSC for both genders and on SR for males, may be explained by the increased fat mass located such that it hinders the range of motion, possibly reflecting females smaller WC. Furthermore, the high contribution of WC to SBE, MPU and both the explosive power tests, is thought to be explained by the weight bearing characteristics of those MSMF tests. The inverse association between increased scores on BMI, WC and body fat percentage and lower scores on MSMF have been reported in previously published studies [6, 14–16]. Fogelholm, Malmberg, Suni, Santtila, Kyrolainen and Mantysaari [14]. clearly state that the functional muscle fitness is impaired in individuals with abdominal obesity, and that the decline in MSMF should be given increased attention.

Muscular strength has previously been found to provide unique and important benefits to the prevention and treatment of cardiovascular disease and mortality in addition to several other health and fitness variables, including the prevention of adiposity gains [1]. Maintaining or improving muscular fitness together with flexibility and balance can be crucial for remaining independent [3]. Thus, there is a clear need to achieve and retain a high MSMF level and to reduce fat mass in order to prevent future functional limitations among adults [2, 3, 14, 15].

### Strengths and limitations

The primary strength of our study is that the studied population is based on a nationally random sample of Norwegians from regions across the entire country. Secondly, objective measuring methods for recording MSMF and obesity were used, and all elements of MSMF and obesity were measured by established measuring procedures.

The present study’s primary limitation is the relatively low participation rate with 32 % of the initially invited sample participating in the initial phase (phase I) of this larger survey [24]. Statistics Norway performed a drop-out analysis, and revealed higher socioeconomic background for those participating in phase I of this study, compared to the non-participating invitees. Comparing educational level between the participants of phase I and phase II of this study reveals similar educational level between participants. As higher socioeconomic status (i.e., educational level, personal income, employment status, and ability to pay for basic needs) has been inversely related to impaired physical fitness [33], the drop-out analysis may indicate that the normative values put forward through this paper possibly overestimates the MSMF in the Norwegian population. Furthermore, summing the OLS test with the OLS blinded test into an OLSsum variable, was meant to prevent the clustering of maximum scores observed in the OLS test and create more normally distributed test scores. The high percentage managing to endure the OLS test in addition to the high percentage ending the OLS blind test within the first 15 s, renders the question of whether or not the OLS or the OLSsum are valid measures of balance as a neuromotor function.

Not adjusting for confounding variables in the regression analysis was done with the intent of displaying crude descriptive data, though this may be a bias to the presentation and interpretation of the results. Moreover, some of the normative values should be interpreted with caution as the presentation of normative values by gender and 10 year age groups, rendered few cases (*n* < 20) in three of the female age groups.

## Conclusions

The present study offers warranted normative data on MSMF based on a national sample primarily Caucasian adult Norwegians, making it possible for others to compare results from various field based MSMF tests to normative data based on age and gender. Furthermore, the results displayed a clear decrease in MSMF test scores with increasing age and with increasing WC. This indicates the need to enhance MSMF in population subgroups with these characteristics, in order to prevent disease and mortality in addition to secure an independent daily lifestyle.

## Additional files

Additional file 1: Appendix 1. Measuring procedures for MSMF. The measuring procedures of the musculoskeletal and motor fitness (MSMF) tests used in the present study. (PDF 194 kb)
Additional file 2: Availability of data. The available data generated or analyzed for this research article. (XLSX 247 kb)

## Abbreviations

BSC: Back scratch test
EPP: Explosive power on a power platform test
HGS: Hand grip strength test
MPU: Modified push-ups test
MPUK: Modified push-ups on knees test
MSMF: Musculoskeletal- and neuromotor fitness
OLS: One leg standing test
SBE: Static back extension test
SR: Sit and reach test
VJ: Vertical jump test
WC: Waist circumference
