# Supplementary material for: Normative values for musculoskeletal- and neuromotor fitness in apparently healthy Norwegian adults and the association with obesity: a cross-sectional study
Source: BMC Sports Sci Med Rehabil. 2016 Nov 18;8:37. doi: 10.1186/s13102-016-0059-4 (PMC5116214; doi:10.1186/s13102-016-0059-4)
Supplement: Additional file 1: Appendix 1. — Measuring procedures for MSMF. The measuring procedures of the musculoskeletal and motor fitness (MSMF) tests used in the present study. (PDF 194 kb) [file 13102_2016_59_MOESM1_ESM.pdf]

MUSCULOSKELETAL FITNESS

Muscular endurance

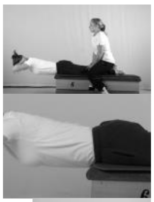

Muscular endurance was measured by the static back extension (SBE) test (Suni, 2000). The participants lay face down on a 15 centimeter (cm) tall, 18 cm broad and 135 cm long bench with their spina iliaca anterior superior lined with the bench's short side, leaving the upper body in a horizontal position for a s long as they could and the time the participants managed to hold a horizontal position was recorded (min 0 seconds (sec), max 240 sec.).

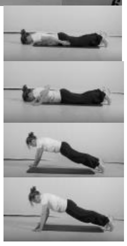

Muscular dynamic endurance and ability to stabilize the upper body was measured by the modified push-ups (MPU) test (Suni, 2000). The participants lay face down on the floor, with their arms alongside their body. The test was initiated by the participants touching the side of their hips, followed by an ordinary push-up with the body held straight. All push-ups where the participants placed the one hand over the other while the albows were extended were counted. Those participants who were not able to perform the MPU with a straight body, performed the test on their knees (MPUK). The number of repetitions completed in 40 sec. were recorded. MPU and MPUK test scores were recored separately.

Muscular strength

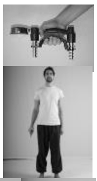

Muscular strength was measured by handgrip strength (HGS) test (Al Snih et al, 2002; Sasaki et al, 2007) using a hydraulic dynamometer (Chattanooga, Hixon, USA). The participants were to stand up straight with their arms hanging down alongside their body, about 10 cm out from the body. The dominant hand held the dynamometer. The best of three attempts was recorded to the nearest 1 kilogram (kg).

Muscular power

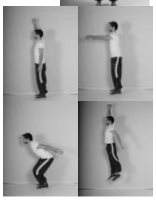

The vertical jump (VJ) test (Suni, 2000) measured explosive power in the lower extremities. The participants were to stand with a foot of choice alongside the wall. With a piece of chalk, the participants reached up the wall, as high as possible, setting a mark on the wall. The participants then stood one foot away from the wall and were instructed to jump as high as possible, setting a mark on the wall with the chalk at peak height of the jump. The distance between the chalk mark set at standing height and that set at peak jumping height was recorded to the nearest cm.

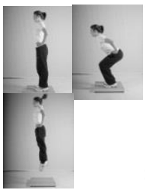

The explosive power (EPP) test measured on a power platform (HurLabs Forceplatform), registered explosive power in the lower extremity. The participants were instructed to stand on the middle of the power platform, the feet placed with a shoulder width distance apart and hands on the hips. The arms were to stay on the hips throughout the jump. The participants were instructed to jump as high as possible. The best of three results was recorded in cm.

Flexibility

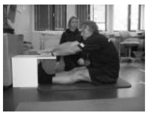

Flexibility of the hamstring musculature was measured by the sit and rach (SR) test (Presidents council on Physical Fitness and Sports, 2008). A specially designed box was placed to a wall and the participants sat on the floor with their knees and upper body straight, and their heels against the box. The participants leant as far along the measuring tape on top of the box as possible, with one hand on top of the other and the back and legs straight. The furthest the participants managed to stretch their hands along the measuring tape and hold for two seconds, was recorded to the nearest half cm. Point zero, the point where the feet met the box was set at 23 cm from the box's edge, and the recorded result was 23 cm plus or minus the distance from point zero.

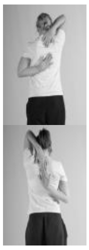

The back scratch (BSC) test (Rikli and Jones, 1999) measured flexibility in the shoulder joint. The participants were standing upright, placing one hand on the lower back, moving it up the participants spine toward their head. The opposite hand was placed on the participants neck, moving it down the spine, aiming to place the long finger of each hand as near each other as possible or to overlap. The procedure was repeated with opposite hands. The gap between the fingertips of the long finger of both hands was measured to the nearest half centimeter. The results were recorded to the nearest half cm, as positive numbers as long as the fingers overlapped and with negative numbers if the fingers did not meet or overlap.

MOTOR FITNESS

Balance

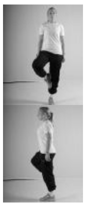

Balance was recorded by means of the one leg standing (OLS) test (Suni, 2000). The participants were instructed to stand on one optional leg, facing a mark at eye height on a wall three meters away. The non-balancing legs heel was to be placed in the knee joint of the supporting leg and the knee was to be rotated externally. The participants arms hung alongside their body. The total time the participants managed to hold the initial balancing position was recorded in sec. If the participants managed to hold the position for 60 sec, the same test procedure was repeated, only blinded. Both test times were recorded separately and summed.
